# Catheter ablation in a geriatric case presenting idiopathic ventricular fibrillation initiated by Purkinje-related premature ventricular contractions

**DOI:** 10.1016/j.hrcr.2024.02.013

**Published:** 2024-02-28

**Authors:** Naoya Kataoka, Teruhiko Imamura, Keisuke Uchida, Takahisa Koi, Koichiro Kinugawa

**Affiliations:** Second Department of Internal Medicine, University of Toyama, Toyama, Japan

**Keywords:** Purkinje, Idiopathic ventricular fibrillation, Short-coupled premature ventricular contractions, Verapamil, Purkinje-muscle junction

**Key Teaching Points**

• The present case exemplified an idiopathic short-coupled variant of ventricular fibrillation (VF) with a new onset at the age of 74 years, requiring catheter ablation, despite the arrhythmia being recognized as an occurrence manifesting before the age of 70.
• The intracardiac electrocardiograms recorded at the left posterior fascicle revealed local muscle-to-Purkinje conduction delay affecting the subsequent R-R interval during polymorphic ventricular tachycardia. The localized conduction block ultimately terminated the arrhythmia.
• This idiopathic VF may onset at an older age than conventionally known, and there is a possibility of involvement of the Purkinje-muscle junction in sustaining the tachyarrhythmias.

## Introduction

Ventricular fibrillation (VF) with no obvious heart disease, as determined by echocardiography, catheterization, or magnetic resonance imaging, termed idiopathic VF, accounts for approximately 10% of cases of aborted sudden cardiac death.^1^ Among them, Brugada syndrome or J-wave syndrome is recognized as subepicardial cardiomyopathy with distinctive electrocardiographic characteristics.^2^

However, a subset of patients with idiopathic VF demonstrate surface electrocardiograms without J-ST-segment elevations, indicating a “normal” electrocardiogram. Short-coupled idiopathic VF has been recognized as a subset of patients exhibiting a “normal” electrocardiogram.^3^ This life-threatening arrhythmia predominantly occurs in middle adulthood, with the age at diagnosis typically reported as less than 70 years in males.^4^ Furthermore, the detailed electrophysiological etiology of this unique arrhythmia remains uncertain.

We encountered a rare case of patients aged over 74 years, involving short-coupled idiopathic VF. The patient exhibited resistance to beta-blocker therapy and subsequently underwent catheter ablation targeting the left posterior fascicle.

## Case report

### Before referral

**Figure 1:**
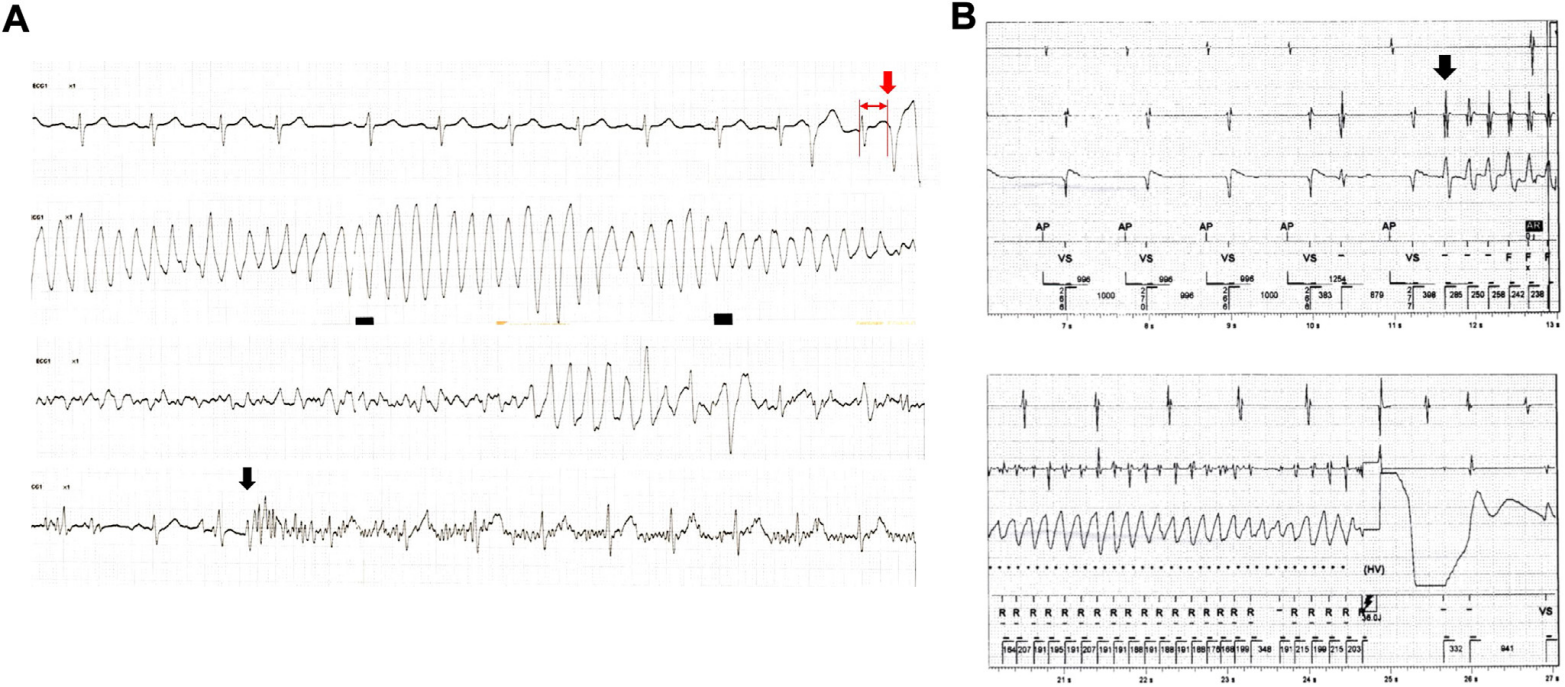
The waveform of ventricular fibrillation initiated by short-coupled premature ventricular contraction (PVC). **A:** The monitored electrocardiogram during the episode of syncope during hospitalization. Ventricular fibrillation occurred initiated by a premature ventricular contraction coupled with a duration of 310 milliseconds (*red arrow*). The noise observed in the bottom line indicates the presence of convulsions (*black arrow*). **B:** Implantable cardioverter-defibrillator recording. The initial PVC morphology exhibited an opposite axis compared to those observed during sinus rhythm (indicated by the *black arrow*). The sweep speed was set at 25 mm per second.

A 74-year-old man presented with recurrent syncope and was hospitalized for the evaluation of the underlying cause. He had a history of diabetes mellitus, dyslipidemia, and a prior cerebral infarction without any neurological sequelae. On the night of day 1, the patient experienced syncope again, accompanied by convulsions. The electrocardiogram monitor showed VF following short-coupled premature ventricular contractions (PVC) (Figure 1A). The physician who conducted the initial examination suspected myocardial ischemia owing to the patient’s past medical history, which included several atherosclerotic risk factors.

### On-admission and in-hospital courses

The patient was referred to our hospital for evaluation with coronary angiography. However, no significant coronary artery stenosis was observed, and the echocardiographic left ventricular ejection fraction demonstrated normalcy, with no abnormal wall motion or wall thinning. There was no evidence of abnormalities related to the mitral valve. The cardiac magnetic resonance imaging also demonstrated normal wall characteristics with no late gadolinium enhancement. The 12-lead electrocardiogram did not reveal the presence of J waves in the inferior or precordial leads, indicative of conditions such as Brugada syndrome or J-wave syndrome. The diagnosis of idiopathic VF was confirmed, and an implantable cardioverter-defibrillator (ICD) was implanted, along with the administration of bisoprolol fumarate 5 mg per day for residual PVC. The patient was discharged 7 days after the initiation of bisoprolol, and no recurrent VF or appropriate ICD therapy was observed for a while.

### VF recurrence and rehospitalization

Nevertheless, appropriate ICD therapy was applied for VF twice within 2 months following the index hospital discharge. The PVC morphology exhibited an opposite axis compared to those observed during sinus rhythm (Figure 1B). Consequently, catheter ablation was undertaken for idiopathic VF following the rehospitalization.

**Figure 2:**
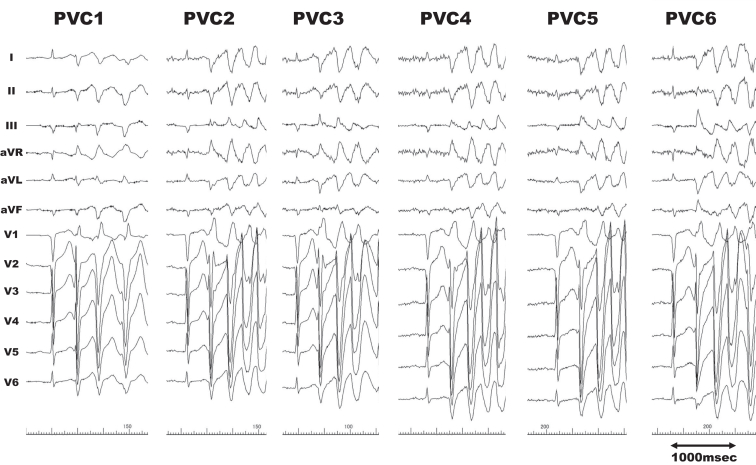
Premature ventricular contractions leading to polymorphic ventricular tachycardia. Six waveforms in total demonstrated the right bundle branch block pattern with varying axis deviations.

The endocardial approach to the left ventricle through transatrial septal puncture with local anesthesia was executed, employing the 3-dimensional mapping system (EnSite X; Abbott, Abbott Park, MN). The PVCs generally exhibited a right bundle branch block pattern with superior axis deviation; however, 6 patterns with different axis leads resulted in polymorphic ventricular tachycardia (PMVT) during the procedure (Figure 2). The average coupling interval of PVCs was 354 milliseconds.

**Figure 3:**
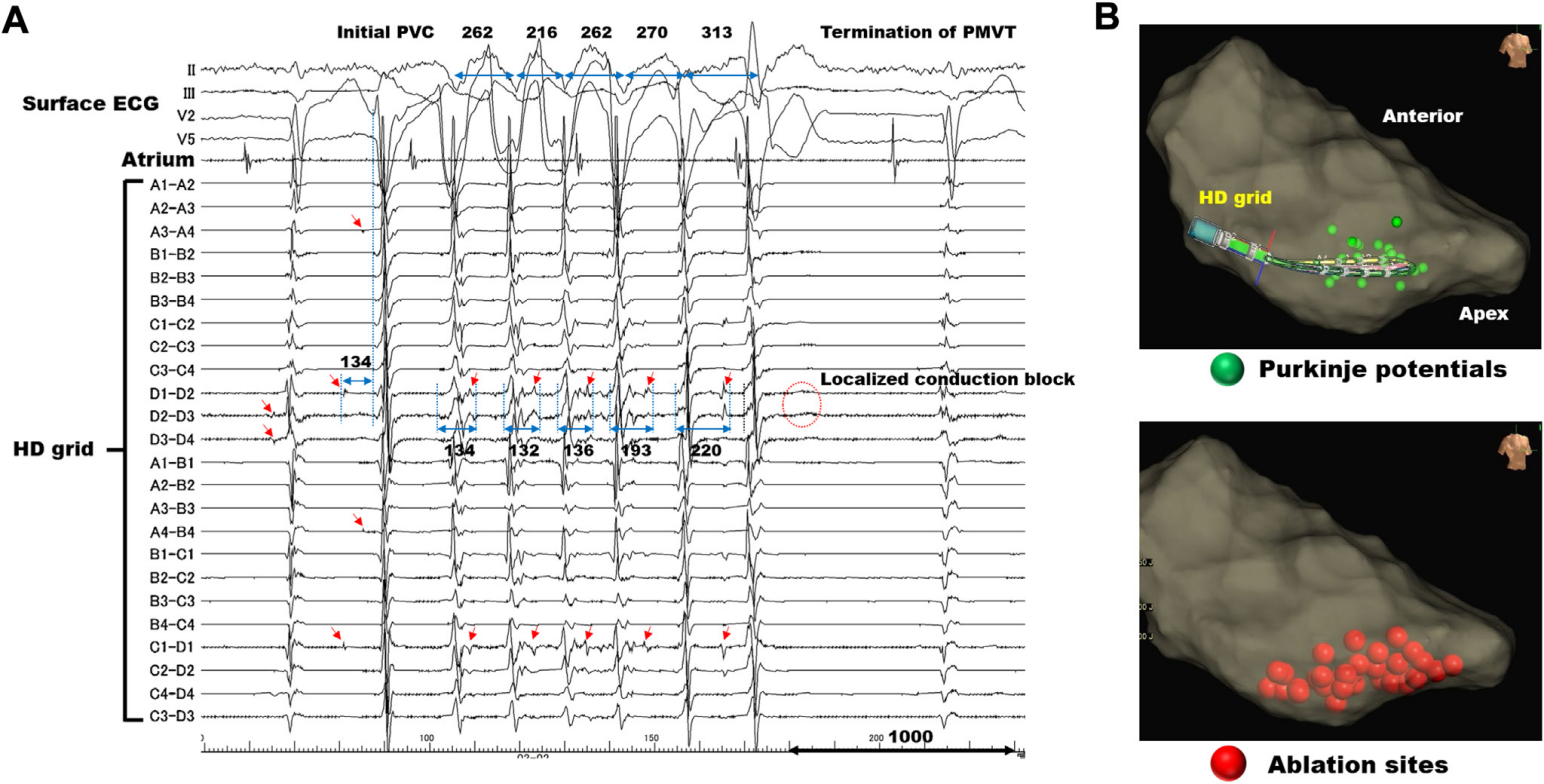
Ablation records. **A:** The fascicular-Purkinje potentials in the left posterior fascicle area exhibited decremental conduction during polymorphic ventricular tachycardia. Ultimately, the localized conduction block of the fascicular-Purkinje potentials led to the termination of the tachycardia. Red arrows indicate the fascicular-Purkinje potentials; blue lines, the distances between R-R or the fascicular-Purkinje potentials and the local myocardial potentials. The values represent intervals, and the unit is milliseconds. **B:** The upper section demonstrates the positioning of the HD grid recording intracardiac electrocardiograms with a 35° right anterior oblique view. Green tags indicate the positions of fascicular-Purkinje potentials, estimated as the left posterior fascicle. The lower section illustrates the ablation site, depicted with red tags.

Given that the waveform of the PVCs suggested an origin located in the left posterior fascicle area, a grid mapping catheter (Advisor HD Grid Mapping Catheter Sensor Enabled; Abbott) was positioned in the inferior-septum region of the left ventricle to anticipate the initial PVCs. Figure 3A illustrates the intracardiac electrocardiograms.

Although several forms of PVCs were observed during the mapping, and some of them could record the pre-excited Purkinje potentials, we were unable to detect the earliest activation site in the fascicular-Purkinje system. This was due to the various coupling intervals between the Purkinje potential and the onset of surface PVCs observed in those PVCs. However, at the site where left posterior fascicle potentials were demonstrated during sinus rhythm (bipolar electrodes D2-D3 and D3-D4 in Figure 3A), fascicular-Purkinje ectopy occurred 134 milliseconds prior to the onset of surface electrocardiogram of the initial PVC leading to PMVT.

Subsequently, delayed fascicular-Purkinje potentials, a subset of which were accompanied by continuous fractionated potentials, were observed following the local myocardial potentials. This observation was confirmed in bipolar electrodes of D1-D2, D2-3, and C1-D1 in Figure 3A. The location where the HD grid mapping catheter recorded the intracardiac electrocardiograms is depicted in Figure 3B.

The conduction interval from the local myocardium to the fascicular-Purkinje system was reflected in the subsequent R-R interval during the tachycardia. Ultimately, the localized conduction block from the local myocardium to the fascicular-Purkinje system led to the termination of PMVT (the red dotted circle in Figure 3A).

The results of these electrophysiological tests confirmed that the abnormal firing of the fascicular-Purkinje system located in the left posterior fascicle was responsible for initiating PVCs, while the Purkinje-muscle junction played a pivotal role in sustaining PMVT.

### Therapeutic catheter ablation

Therefore, radiofrequency ablation for the left posterior fascicle area, especially at the location where abnormal fascicular-Purkinje ectopies were confirmed, was performed with the aim of ablating the Purkinje-muscle junction (Figure 3B). Although achieving complete suppression of PVCs posed a challenge, the blockage of the left posterior fascicle occurred during ablation, resulting in the disappearance of PVCs. After confirmation of the disappearance of spontaneous PVCs with the isoproterenol provocation test or ventricular programmed stimulation, the procedure was concluded.

### Following discharge

Two days after the discharge, spontaneously terminating PMVT was confirmed in the ICD record. The initial PVC could not be detected on the surface electrocardiogram; however, the detected morphology in the ICD record underwent a change compared to the index PVCs demonstrated in Figure 1. This might suggest that the PVC originated from a different region than the ablation site. Subsequently, oral administration of verapamil at a daily dose of 120 mg was initiated, and no recurrence of VF was observed through home monitoring of the ICD during the subsequent 2 months thereafter.

## Discussion

The present case highlights 2 novel insights. Firstly, the onset age was notably higher compared to prior investigations into idiopathic VF, estimated to be the highest one among the literature. Secondly, the electrophysiologic findings provided evidence of the contribution of abnormal excitability of the fascicular-Purkinje system to PVCs and the involvement of the Purkinje-muscle junction in sustaining PMVT.

### Age of new-onset and PVC characteristics

The latest reviews indicated that the prevalent age for the onset of idiopathic VF triggered by R-on-T-type PVCs was under 70 years old; additionally, only 1 female patient over 70 years old was observed.^4^ The present case represented the second instance of new-onset idiopathic VF in an individual over 70 years old, and the first occurrence in a male patient.

The literature review reported that approximately half of the patients experience a recurrence of syncope; similarly, this case also presented a recurrence of syncope.^4^ Although more than 70% of patients exhibit the left bundle branch block pattern PVCs, this case demonstrated the right bundle branch block pattern.^4^

The mean coupling intervals of PVCs have been reported as 293 ± 50.5 milliseconds, and coupling intervals less than 350 milliseconds are considered as “short” triggering PMVT or VF.^4^ This case demonstrated a mean coupling interval of 354 milliseconds, often referred to as “not-so-short.” Although we previously reported a case of multifocal PVCs originating from both ventricles, the present case exhibited PVCs only from the left ventricle with various axis patterns.^5^ This phenomenon suggested that the hyperexcitability of the left bundle fascicular-Purkinje system facilitates spontaneous PVCs, and the excitation of the working myocardium at an exit site occurred at multiple locations through the Purkinje network.

### Significance of the Purkinje-muscle junction for PMVT/VF

Purkinje-muscle reentry has been established as a potential mechanism of polymorphic ventricular arrhythmias, including PMVT and VF.^6^^,^^7^ From the computerized 3-dimensional model, reentry activities were observed from Purkinje fibers to the muscle, or in the opposite direction, at the stage of PMVT before transitioning to VF.^6^ The involvement of the impaired Purkinje network in the reentry circuit leading to VF was demonstrated in a case of myocardial infarction.^8^ These results suggest that the Purkinje-muscle junction plays a critical role in sustaining PMVT. Our findings also demonstrated that local Purkinje activities followed local myocardial activities during the PMVT, and the conduction intervals between local muscle and Purkinje fibers influenced the subsequent R-R intervals.

Additionally, the termination of PMVT was observed with the conduction block from muscle to Purkinje fibers (Figure 3A). These electrophysiological findings suggest that the Purkinje-muscle junction plays a significant role in maintaining polymorphic ventricular arrhythmias, even in the absence of discernible underlying etiologies, such as ischemia. Recent papers reported the observation of VF initiated by Purkinje-related PVCs in inferolateral J-wave syndrome.^9^^,^^10^ Nademanee and colleagues^10^ proposed that the mechanisms of VF be classified into 2 categories: 1 involving conduction abnormalities as mechanisms to sustain VF, and the other associated with Purkinje abnormal ectopies related to VF triggers. In our previous paper on Brugada syndrome, we regarded phase 2 reentry as the mechanism for triggered PVCs, and conduction abnormalities as the mechanisms sustaining VF, categorizing as the former.^11^ However, the present case, which exhibited no J-ST elevation in surface electrocardiograms, was considered to be categorized as the latter.

In terms of ablation technique, we opted for the strategy of ablating the Purkinje-muscle junction in the area of the left posterior fascicle, despite being unable to detect the earliest activation site in the fascicular-Purkinje system of spontaneous PVCs. Although this strategy resulted in a left posterior fascicle block, sustained PMVT or VF requiring ICD shock therapy was no longer observed. This ablation technique, involving the ablation of the fascicular-Purkinje network, is recently referred to as “de-networking,” which typically refers to linear ablation for the transection of the left posterior fascicle.^12^ However, the strategy might produce results of suppressed VF recurrences but persist with the initial PVCs.

Regarding antiarrhythmic therapies, quinidine has been recognized as one of the most effective antiarrhythmic drugs for idiopathic VF.^4^ In light of the supply instabilities of quinidine in Japan, bisoprolol or verapamil was administered in this case to mitigate the initial PVCs or recurrence of PMVT/VF. In the event of future recurrences of VF, consideration should be given to administering quinidine.

## Conclusion

The present case showcased idiopathic VF with a new-onset age higher than that reported in previous studies, requiring repeated ICD shock therapy. Successful suppression was attained by catheter ablation targeting the fascicular-Purkinje system along with the administration of verapamil. The conduction at the Purkinje-muscle junction is expected to play a role in sustaining PMVT/VF. Although verapamil might primarily contribute to suppressing the initial PVCs, catheter ablation must have contributed to shortening the duration of PMVT/VF.
